# Delayed presentation of anorectal malformations

**DOI:** 10.4103/0971-9261.43023

**Published:** 2008

**Authors:** Shandip Kumar Sinha, Ravi P. Kanojia, Ashish Wakhlu, J. D. Rawat, S. N. Kureel, R. K. Tandon

**Affiliations:** Department of Pediatric Surgery, King George Medical University, Lucknow, India

**Keywords:** Anorectal malformation, delayed presentation

## Abstract

**Aims and Objectives:**

Delayed management of anorectal malformation (ARM) increases the surgical and functional complications for the patient. We defined “delayed presentation of ARM” and reviewed our patients with ARM to find out the incidence and causes of delayed presentation.

**Materials and Methods:**

Patients satisfying the criteria of “delayed presentation of ARM” were involved. Detailed information of each patient including the mode of presentation, associated anomalies, plan of management and follow-up was obtained from the hospital records.

**Results:**

Between 2003 and 2006, 43 patients satisfied our criteria of “delayed presentation of ARM”. There were 21 males and 22 females. Seventeen of these males presented with low-type ARM. Eleven of them were managed by a single-stage procedure. These “delayed presenters” had to live with constipation, inadequate weight gain and parental anxiety for a greater time. Analysis of the outcomes showed more functional complications in patients who had undergone failed perineal surgery previously. In females with low ARM, the procedure of choice was anterior sagittal anorectoplasty (ASARP). Single stage surgery provides good outcomes for most of low type of ARMs. High-type ARMs in males and females were managed by a staged procedure.

**Conclusion:**

“Delayed presentation of ARM” is a major group of ARM in our setup. The management and results of their treatment are not different from those of the early presenters. The most common cause of delayed ARM is wrong advice given by the health care providers followed by inadequate treatment elsewhere. Corrective surgeries taking second attempt in perineum always produces poor outcomes.

## INTRODUCTION

Anorectal malformations (ARMs) are one of the most common congenital anomalies dealt by pediatric surgeons. They range in severity from imperforate anal membrane to complete caudal regression. Although majority of the anomalies are detected in the neonatal period, there are a significant number of patients who report at a later age. Delayed management increases surgical and functional complications for the patient. We reviewed our patients with ARMs to find out the incidence and causes of delayed presentation. This paper describes the anomalies and management of patients with delayed presentation of ARM.

## MATERIALS AND METHODS

**Figure 1:**
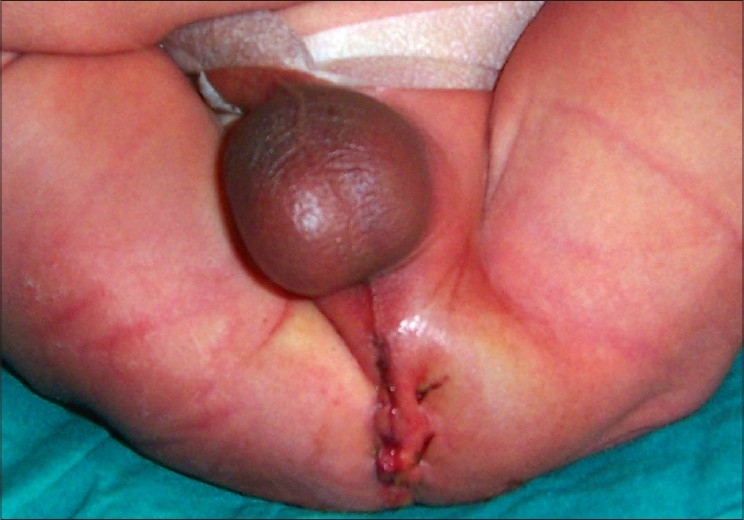
A newborn with an unsuccessful perineal attempt to repair the ARM: “Dug perineum”

All the patients with ARM who reported between August 2003 and August 2006 at the King George Medical University Hospital were identified from the medical records. This is a tertiary referral center serving a population of over 30 million people. “Delayed presentation of ARM” refers to a patient who presented after 7 days of birth, except for a female patient, with low-type ARM, where presentation beyond 6 months of age was considered as delayed. Inadequately treated patients were also considered as “delayed presentation of ARM”. The term “dug perineum” was used to describe the patients with ARM, who had undergone unsuccessful perineal attempts for the repair of the malformation [Figure 1]. Detailed information regarding each patient including the mode of presentation, associated anomalies, plan of management and follow-up was obtained from the hospital records.

## RESULTS

During the study period, 171 patients with ARM were treated in our department. Forty-three of these satisfied our criteria of “delayed presentation of ARM” and were included in this study. Thirty-eight of these patients were born by normal vaginal delivery at home, and five were born at a primary health centre. There were 21 males and 22 females, varying in age from 7 days to 19 years. On the analysis of the type of anomaly, it was found that 11 patients had high-type ARM (27.5%) [Table 1]. The average weight of these patients was 7.1 kg (expected weight - 10.7 kg). For clarity of description, the male and female patients are considered separately.

**Table 1:** Details of delayed presentation of anorectal malformation

	High	Low type	Associated anomaly	Dug perineum
Male	4	17	3	6
Female	7	15	4	0

### Male patients

The age of presentation of males ranged from seven days to five years. It was found that 17/21 (81%) of the male patients had low-type ARM.

#### Low-type ARM (male)

Fifteen out of 17 males with low-type ARM presented within the first year of life. The causes for their delayed presentation were the delayed detection of the problem (5), wrong advice regarding treatment (4), inadequate treatment elsewhere (5) and social reasons (2). Only one child of this group presented with constipation. The diagnosis of the low-type ARM was done by clinical examination of perineum. Fecal discharge from an abnormal opening in the perineum, perineal excoriation and scar of previous surgery were visible. Three patients had a misdiagnosis and proximal colostomy done elsewhere. In these cases, distal loopogram was done and this showed the absence of fistula and low-type ARM. Out of the five cases with inadequate perineal procedure, four had colostomy performed by the operating surgeon. All patients were investigated for associated anomalies that were present in three patients. One patient had associated hypospadias, one had undescended testes and the last one had congenital heart disease.

Analysis of the offered treatment showed that single-stage Posterior ‘V’ flap anoplasty was performed in twelve patients including the three who had previous colostomy. Two patients had “cutback anoplasty” and one patient had anterior sagittal anorectoplasty (ASARP) done. One child was managed by initial colostomy followed by ASARP because of perineal excoriation following dug perineum. One patient could not be operated because of his cardiac failure [Table 2]. All patients undergoing anoplasty had bowel preparation with normal saline 6 h before the surgery. After anoplasty, oral feeds were started in the evening. They were discharged the next morning with instructions for a sitz bath and oral lactulose twice a day and were advised to report after 7 days and subsequently every month. Size of the anal opening, anal tone and bowel habits were assessed during the subsequent visits. Three of the 15 patients required regular dilatation. All patients had good anal tone. In patients with colostomy (7/17), closure was done after 3 months. One patient has severe constipation for which regular enema and lactulose is advised. Three patients (dug perineum patients) have occasional soiling.

**Table 2:** Delayed presentation of anorectal malformation in males

	No. of patients	Treatment	Dug perineum
ARM (high)	4	Colostomy - 3	0
		Abdomino PSARP - 1	1
ARM (low)	17	Colostomy - 1	1
		ASARP - 1	1
		Cutback anoplasty - 2	0
		Barrow flap anoplasty - 12	3

ARM - Anorectal malformation

Review of our data also showed that this group of patients had the maximum incidence of inadequate treatment. Inadequate perineal surgery was done in five patients, necessitating colostomy in four. Only one patient with inadequate perineal surgery had single stage anoplasty. These patients had difficult anoplasty because of dense fibrosis and loss of tissue planes. There was an intraoperative urethral injury in one of the patients with second perineal surgery, in which ASARP was done. Postoperatively, three of these five patients had occasional soiling.

#### High-type ARM (male)

Four out of 21 cases of the “delayed presentation of ARM” male patients had high-type ARM and were passing stool through the urethra. All of these were delivered at home with uneducated parents. Lack of information regarding the availability of treatment was the cause of delayed presentation in three patients, who reported after 2 weeks of birth. The diagnosis was confirmed by history of passage of stool with urine and the absence of anal opening on clinical examination. There were no associated anomalies in this group. Sigmoid colostomy was carried out in all the cases. Inadequate treatment was the cause of delayed presentation in the last male patient, who presented at the 9 months of age. He had history of perineal exploration elsewhere. He was a case of congenital partial short colon and abdomino-posterior sagittal anorectoplasty (PSARP) with ileostomy was carried out.

### Female patients

Twenty-two female patients with ARM satisfying our definition of “delayed presentation of ARM” were admitted during this period. Seven of them had high-type ARM.

#### Low-type ARM (female)

**Figure 2:**
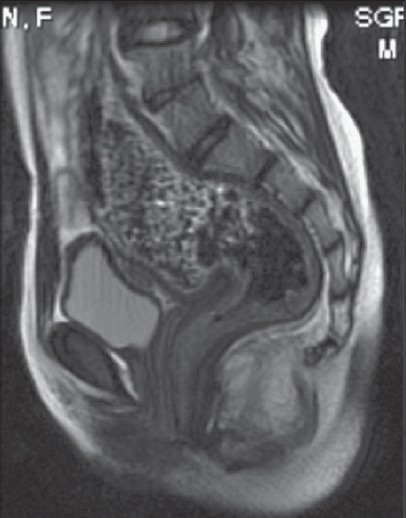
MRI picture of an 11-year-old girl with anovestibular fistula

All the girls with low-type ARMs were passing stool from abnormal proximal opening. Eleven of these were born at home and four were delivered at a primary health centre. The most common cause of their delayed presentation was wrong advice regarding the age of treatment (5), delayed knowledge of problem (4), constipation (3) and social reasons (3). None of the girls had been operated elsewhere. Three girls with low ARM presented after 10 years of age [Figure 2]. One of them presented to a gynecologist for infertility and during the evaluation anovestibular fistula (AVF) was diagnosed. The diagnosis in all the female patients with low-type ARM was confirmed by clinical examination. There were seven cases with AVFs, three cases with anterior ectopic anus (AEA), three cases with rectovestibular fistula (RVF), one case with perineal canal and one case with anal stenosis. There were two girls with associated anomalies in this group; in both cases, the vagina was absent (Mayer Rokitansky syndrome). All the patients were managed by single-stage ASARP under general anesthetics. Preoperative distal bowel wash was conducted the night before surgery. Postoperatively oral intake was started in the evening and the patients were discharged on sitz bath and oral lactulose. Analysis of the postoperative results showed that only one of these patients (RVF) had postoperative wound infection, which healed with dressing and antibiotics. Four of the 15 girls had postoperative constipation and were advised to administer stool softeners. Regular anal dilatation was required in five girls for 3 months. None of the girls reported fecal soiling [Table 3].

**Table 3:** Delayed presentation of anorectal malformation in females

	No. of patients	Treatment	Dug perineum
ARM	Cloaca - 5	Colostomy - 5	0
(high)	Rectovaginal fistula - 2	Colostomy - 2	
ARM	Anal stenosis - 1	Colostomy - 0	0
(low)	Anovestibular fistula - 7	ASARP - 15	0
	Anterior ectopic anus - 3	Cutback anoplasty - 0	0
	Rectovestibular fistula - 3		
	Perineal canal - 1		

ARM - Anorectal malformation

#### High-type ARM (female)

There were seven females with high anorectal malformations that were considered as the “delayed presentation of ARM”. All of them were delivered at home and the female delivery attendant noticed the absence of anal opening in five of them. The causes of their delayed presentation were social reasons (2), delayed advice regarding the availability of treatment (2), difficulty in passing stool with abdominal distention (2) and inadequate treatment (1). Associated anomalies were present in two of these patients. The left kidney was absent in one of them and the other had sacral agenesis. The actual malformation was confirmed after examination under anesthesia and girls had a preliminary colostomy. Second stage surgery has been performed in two girls with rectovaginal fistula and two with common cloaca. The other patients awaited definitive operation.

## DISCUSSION

Anorectal malformations are correctable congenital malformations with reasonably good prognosis. Most of these anomalies can be easily identified by clinical examination in the newborn children and presently the treatment is standardized. For high-type anomalies in both males and females, colostomy is recommended at birth.[1,2] For male patients with low-type malformation, single stage anoplasty is recommended at birth.[3] For female patients with low-type malformations, the current trend is to correct these malformations at presentation, i.e., in the period between birth and six months of age. Pena has reported the operation of children around 2-3 months of age.[4] Others have recommended such corrective procedures soon after birth, thereby expecting to achieve optimal results with lesser complications.[5] Safer anesthesia techniques, better monitoring during surgery and technical expertise have probably lead to the consideration of such procedures. We recommend these patients to be operated within 6 months of age and consider the older patients as “delayed presenters”.

Delay in diagnosis can have an adverse impact on the management of these malformations. Developed countries have occasional case reports of delayed presentations of these malformations,[6–8] but such presentations have a high incidence in our state. Our study shows that about 25% of ARMs are “delayed presenters”.

The analysis of the causes of delayed presentation shows that wrong advice regarding the correct age of treatment of these malformations is the most common cause. This may be because most of the cases with “delayed presentation of ARM” have low-type anomalies and the child is passing stool. Therefore, the health workers and family advise them to seek treatment when the mother is able to go with the newborn. The second important cause of delayed presentation is the inadequate management of the ARM elsewhere. All of these patients were males. Incorrect reading of the level of malformation on an invertogram by surgeons leads to misdiagnosis and erroneous attempts for perineal surgery in a malformation that requires colostomy. These cases with “dug perineum” have more surgical complications. Moreover, the damage of the external sphincter leads to an increased incidence of soiling in such low-type ARMs. Delayed diagnosis is important in patients with subtle variety of ARMs such as anterior ectopic anus. Constipation was the mode of presentation in few of the high-type anomalies in females. Social factors such as the lack of money, migrated father working abroad and lack of social support were also the important causes for delayed presentation. There are case reports in the literature[9,10] regarding the presentation of ARM in females at adolescence. In our study, three females with low ARM presented at adolescence because of their proposed marriage. One female with low malformation was diagnosed during the evaluation for infertility [Table 4]. The modes of delayed presentation in our setup are different from developed countries, where constipation and abnormally positioned anal opening detected by parents are more common.[11]

**Table 4:** Causes of delayed presentation

	Males	Females
Inadequate treatment	6	1
Constipation	1	5
Wrong treatment advise	7	7
Delayed diagnosis	5	4
Social factors	2	5

From our fairly large experience with delayed presentation of ARM, we have developed a protocol for the diagnosis and management of such patients. The diagnosis is first confirmed by clinical examination and if necessary by contrast X-ray. The anomaly is then determined. The diagnosis of ARM with delayed presentation requires a similar approach as that required for a neonate. In majority of cases, clinical examination will suffice. In some cases, a fistulogram or distal loopogram is required.

For both male and female patients who present late with low-type ARMs, we recommend ASARP. The advantages are that it is a single-stage procedure and does not require the withholding of oral intake. For high anomalies, colostomy followed by posterior sagittal ano-rectoplasty (PSARP) is recommended. Similar approach for these malformations has been used by many surgeons for patients who presented at birth.[2] Both staged procedures[3,11] and single-stage surgery[12] are recommended for low-type anomalies by the anterior sagittal and posterior sagittal route.[12,13] Treatment has been performed with good outcomes via the posterior sagittal route in a single stage in both the males and females with low-type ARMs.[14] However, these patients required extreme preoperative and postoperative measures, namely, total bowel preparation, postoperative complete bed rest and total parental nutrition (TPN).

Patients with low ARM who had undergone previous attempts at perineal surgery were a unique category. When perineal digging failed, the surgeon had performed colostomy. The definitive procedure that was performed was either ASARP or Posterior ‘V’ flap anoplasty and colostomy closed at a subsequent procedure.

Single-stage ASARP in female and Posterior ‘V’ flap anoplasty in male were the most common surgeries performed. Postoperatively, only one of these patients had a wound infection. Incidence of soiling is more in these patients (50%).

Following the completion of all stages of definitive treatment (30/42), constipation was the most common immediate complication in both males and females, which was managed with stool softeners and enema. Regular dilatation was also required in 25% of these patients. Five patients had anal excoriation, which healed with time. In patients who were less than three years of age, assessment of social continence is not possible.[15] Eleven out of 30 patients with low-type ARM were more than 3 years of age and three (27%) of them showed occasional soiling. Two patients (18%) required laxatives for their constipation.

## CONCLUSION

“Delayed presentation of ARM” are a major group of ARM in our setup. The management and outcomes of their treatment are not different from early presenters. These “delayed presenters” have to live with constipation, inadequate weight gain and parental anxiety for a greater time. Single-stage surgery provides good outcomes for most of the low-type ARMs. Second perineal surgery produces a poor outcome. Protocol-based management provides a greater chance for normal quality of life.
